# Transcriptome changes of liver fluke *Opisthorchis viverrini* in diabetic hamsters[Fn FN1]

**DOI:** 10.1051/parasite/2024056

**Published:** 2024-09-13

**Authors:** Apisit Chaidee, Naruechar Charoenram, Chatchawan Sengthong, Rungtiwa Dangtakot, Porntip Pinlaor, Thatsanapong Pongking, Somchai Pinlaor

**Affiliations:** 1 Department of Parasitology, Faculty of Medicine, Khon Kaen University Khon Kaen 40002 Thailand; 2 Cholangiocarcinoma Research Institute, Khon Kaen University Khon Kaen 40002 Thailand; 3 Institute for Urban Disease Control and Prevention, Department of Disease Control, Ministry of Public Health Bangkok 10220 Thailand; 4 Department of Medical Technology, Faculty of Allied Health Sciences, Nakhonratchasima College Nakhon Ratchasima 30000 Thailand; 5 Centre for Research and Development of Medical Diagnostic Laboratories, Faculty of Associated Medical Sciences, Khon Kaen University Khon Kaen 40002 Thailand; 6 Biomedical Sciences Program, Graduate School, Khon Kaen University Khon Kaen 40002 Thailand

**Keywords:** *Opisthorchis viverrini*, Diabetes mellitus, Transcriptomics, Host-parasite interactions, Liver fluke pathogenesis

## Abstract

A recent study in hamsters showed that infection with the liver fluke *Opisthorchis viverrini* in diabetic hosts worsens the severity of hepatobiliary disease. However, the effects of diabetes on the worm’s phenotype and gene expression pattern remain unknown. This study investigated the impact of diabetes on the global gene expression and development of *O. viverrini* in diabetic hamsters. Parasitological parameters were assessed, and mRNA sequencing with bioinformatic analysis was performed. The study revealed that worm establishment rates in diabetic hamsters were directly correlated with fasting plasma glucose levels. Interestingly, worms collected from diabetic hosts exhibited stunted growth and reduced egg production. Transcriptomic analysis revealed significant alterations in gene expression, with 4314 and 567 differentially expressed genes at 21- and 35-days post-infection, respectively. Gene ontology enrichment analysis highlighted changes in biological processes related to stress response, metabolism, and cellular organization. Notably, genes associated with parasite virulence, including granulin, tetraspanins, and thioredoxins, showed significant upregulation in diabetic hosts. These findings demonstrate the profound impact of host diabetic status on *O. viverrini* development and gene expression, providing insights into the complex interplay between host metabolism and parasite biology, including molecular adaptations of *O. viverrini* in hosts. This study contributes to our understanding of opisthorchiasis in the context of metabolic disorders and may inform future strategies for disease management in diabetic human populations.

## Abstract (fr)

Une étude récente sur les hamsters a montré que l’infection par la douve du foie *Opisthorchis viverrini* chez les hôtes diabétiques aggrave la gravité de la maladie hépatobiliaire. Cependant, les effets du diabète sur le phénotype et le profil d’expression génétique du ver restent inconnus. Cette étude a examiné l’impact du diabète sur l’expression génétique globale et le développement d’*O. viverrini* chez les hamsters diabétiques. Les paramètres parasitologiques ont été évalués et un séquençage de l’ARNm avec analyse bioinformatique a été effectué. L’étude a révélé que les taux d’établissement des vers chez les hamsters diabétiques étaient directement corrélés au taux de glucose plasmatique à jeun. Il est intéressant de noter que les vers récupérés auprès d’hôtes diabétiques présentaient une croissance retardée et une production d’œufs réduite. L’analyse transcriptomique a révélé des altérations significatives de l’expression génétique, avec 4 314 et 567 gènes exprimés de manière différentielle à 21 et 35 jours après l’infection, respectivement. L’analyse d’enrichissement de l’ontologie génétique a mis en évidence des changements dans les processus biologiques liés à la réponse au stress, au métabolisme et à l’organisation cellulaire. Notamment, les gènes associés à la virulence du parasite, en particulier la granuline, les tétraspanines et les thiorédoxines, ont montré une régulation positive significative chez les hôtes diabétiques. Ces résultats démontrent l’impact profond du statut diabétique de l’hôte sur le développement et l’expression génétique d’*O. viverrini*, offrant un aperçu de l’interaction complexe entre le métabolisme de l’hôte et la biologie du parasite, y compris les adaptations moléculaires d’*O. viverrini* chez les hôtes. Cette étude contribue à notre compréhension de l’opisthorchiase dans le contexte des troubles métaboliques et peut éclairer les futures stratégies de gestion de la maladie pour les populations humaines diabétiques.

## Introduction

Opisthorchiasis, caused by the liver fluke *Opisthorchis viverrini*, is a major public health concern in the Greater Mekong Subregion, affecting an estimated 6 million individuals [40]. This infection is associated with the development of hepatobiliary diseases, including cholangiocarcinoma [40]. Established knowledge suggests that *O. viverrini* infection triggers inflammation, fibrosis, and proliferation of the biliary tree [4, 50]. The severity of these pathological changes is known to be influenced by various factors, including the intensity of infection. Additionally, research has demonstrated that host health status can also play a role in disease severity. For instance, studies have shown an increased disease burden in prednisolone-treated and diabetic hamsters [6, 20, 21]. However, limited information exists regarding the impact of altered host health on the parasite itself.

Diabetes mellitus (DM), a chronic metabolic disorder characterized by hyperglycemia, is a significant global health issue. While the complete pathophysiology of DM remains under investigation, the typical underlying cause of hyperglycemia in mammals involves three key defects: impaired insulin sensitivity in peripheral tissues, increased hepatic gluconeogenesis, and compromized insulin secretion. These defects are believed to be a consequence of chronic, low-grade pro-inflammatory responses leading to a loss of functional beta-cell mass. This mechanism is considered the common thread in both type 1 and type 2 diabetes [3, 29]. DM exerts a multifaceted impact on the host, including compromising the immune response and antibody production (thereby increasing susceptibility to microbial infections), altering fatty acid synthesis and deposition, and promoting complications such as heart disease, stroke, and kidney disease [45].

The interplay between parasitic infections and the diabetic host is an emerging area of research. Interestingly, some studies suggest that parasitic infections might have the potential to prevent or reduce the severity of diabetes, opening avenues for novel therapeutic strategies for both conditions [30]. Furthermore, it is well-established that the health status of the host can significantly influence the growth, development, and establishment rate of various parasitic species, encompassing both protozoa and helminths. Examples include *Echinococcus granulosus*, *Enterobius vermicularis*, *Schistosoma mansoni* and *S. haematobium*, *Hymenolepis nana*, hookworms, *Taenia* species, *Giardia lamblia*, *Entamoeba histolytica*, and *Cryptosporidium* species [51]. This influence likely stems from alterations in host immunity, fatty acid and cholesterol profiles, and the parasite’s dependence on the host for essential nutrients and survival factors [1]. Notably, adult worms may also rely on host insulin, which plays a crucial role in numerous biological processes, including glucose uptake, parasite growth, and metabolism. Therefore, the absence of host insulin might impact worm growth and reproduction [46]. We hypothesize that, similar to other parasites, the growth and development of *O. viverrini* within its definitive host are entirely dependent on host-derived nutrients, representing a critical factor in the parasite’s biology and ecology. By gaining a deeper understanding of this intricate relationship, we can develop more effective strategies for controlling opisthorchiasis infections.

This study aimed to investigate the impact of DM in the host on the growth and development of *O. viverrini*. We report on the parasitological changes in the adult worm and utilize transcriptomics analysis to demonstrate the global changes in gene expression of the worm that developed in a diabetic host.

## Materials and methods

### Ethics

The study protocol was approved by The Animal Ethics Committee of Khon Kaen University, based on the ethical guidelines for Animal Experimentation of the National Research Council of Thailand, Ref.no. KKUAE 21/2556 and IACUC-KKU-46/67.

### Animals and induction of diabetes

Sixteen males 6- to 8-week-old Syrian golden hamsters, *Mesocricetus auratus*, average body weight of 140 g, were obtained from the Animal Unit of the Faculty of Medicine, Khon Kaen University, maintained under a standard light cycle (12 h dark/light) and provided with *ad libitum* access to water and food (Smart heart, Thailand). Hamsters were assigned into 2 groups: normal control (NC) (*N* = 6) and streptozotocin (STZ)-induced diabetes (DM) (*N* = 10). Streptozotocin, an antineoplastic agent that is toxic to the insulin-producing beta cells of the pancreas, was used to diabetic induction as previously described [6, 36]. Diabetes was induced using intraperitoneal injection of 40 mg/kg body weight of STZ dissolved in 0.1 M tri-sodium citrate buffer, pH 4.5 for 3 consecutive days. Hamsters in the NC group received sodium citrate buffer alone. Only hamsters with fasting blood glucose (FBG) > 250 mg/dL at two weeks post-injection were selected as DM hamsters.

### *Opisthorchis viverrini*: infection and specimen collections

*Opisthorchis viverrini* metacercariae were obtained from naturally infected cyprinid fish [32]. Hamsters were infected by 50 alive metacercariae as observed by larval movement under light microscope, by gastric intubation [37]. Metacercariae that did not exhibit larval movements were discarded. Hamsters in the DM group were infected with metacercariae at 4 weeks after induction of diabetes.

**Figure 1:**
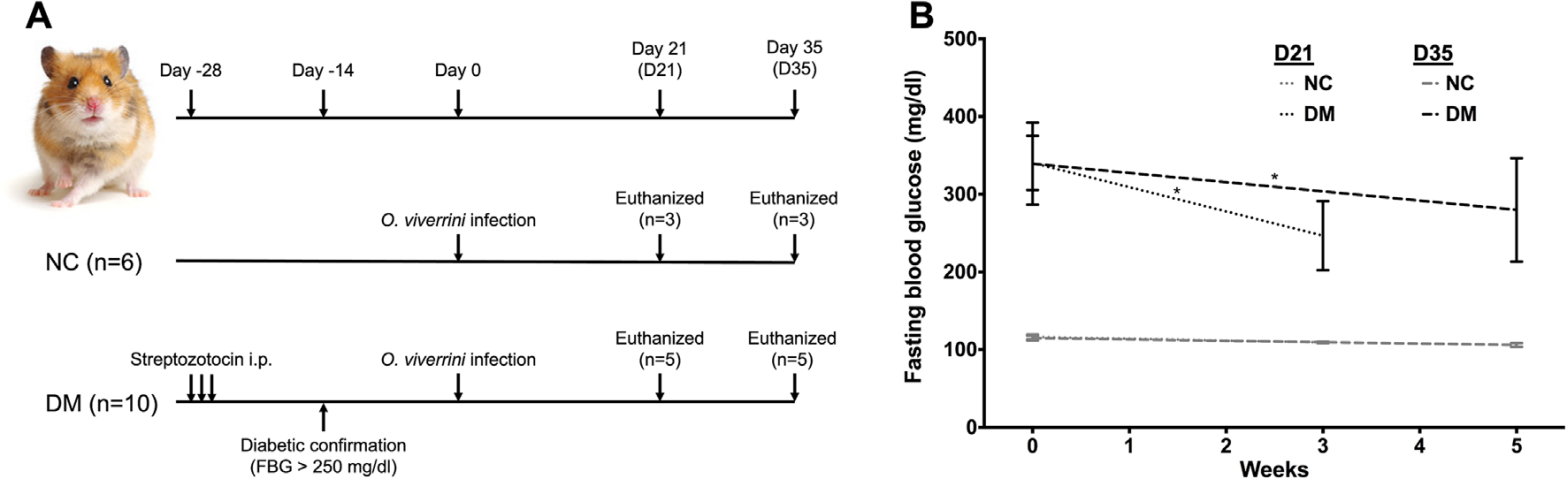
Experimental plan of the study (A) and the fasting blood glucose of the hamsters measured in normal (NC) and diabetes (DM) groups, pre- and post-infection (B). ^*^p < 0.05. FBG: Fasting blood glucose.

Figure 1A shows the experimental plan. Hamsters were euthanized at designated time points: 3 and 5 weeks post-infection (D21 and D35, respectively). Prior to euthanasia, hamsters were food deprived for 2 days. Hamster was euthanized with overdose diethyl ether. At necropsy, blood, feces, and worms were collected. Blood glucose levels were measured using a glucometer (Accu-Chek Advantage II; Roche Diagnostics, Basel, Switzerland). Worms were squeezed out from the liver under normal saline solution and washed with phosphate buffered saline (PBS). Released worms were counted and the percent worm establishment rate was calculated. All worms were separated into 2 batches, as follows: first, for a parasitological study, worms were paralyzed using warm distilled water and warm formalin. They were then kept in 70% ethyl alcohol until use. Second, for a molecular biology study, worms were placed in Trizol reagent (Thermo Fisher Scientific, Waltham, MA, USA) and snap frozen using liquid nitrogen. Worms in Trizol and fresh feces were stored at −80 °C prior to use.

### Parasitological study

#### Worm staining

To observe worm morphology, worms were stained with aceto-alum carmine, as previously described [20]. Stained *O. viverrini* adult worms were observed for body size, reproductive organ size, and other physical aspects using a dissecting microscope [20].

#### Reproductive system studies: eggs per worm and fecal egg count

To examine the maturity of the reproductive organ, the number of eggs per worm was examined. Five adult worms from each hamster were randomized, placed in 70% ethanol, and individually crushed with a mortar. The solution was then collected and adjusted to 5 mL by adding 70% ethanol. The number of eggs was counted by smearing 50 μL of the solution on a glass slide. The experiment was performed in duplicate, and the results are shown as the average number of eggs per worm, which was calculated as follows:

Eggs per worm = (Number of counted eggs × 5)/0.05

The amount of *O. viverrini* eggs in feces was determined using the modified formalin-ethyl acetate concentration technique, as previously described [2]. Two grams of feces sample were used. The number of eggs was counted and presented as the number of eggs per gram (EPG) of feces.

### Total DNA extraction

Worms were weighed to approximately 15–25 mg and placed into a 1.5 mL sterile microcentrifuge tube. Then, genomic DNA (gDNA) was extracted using a QIAamp Tissue Kit (QIAGEN, Hilden, Germany), according to the manufacturer’s protocol. Concentration, purity, and integrity of the gDNA were determined by spectrophotometry (Nanodrop 2000; NanoDrop Technologies, Wilmington, DE, USA) and the quality was checked by electrophoresis on 1.5% agarose gel. The gDNAs were stored at −80 °C for further study.

### ELISA for 5′-methylcytosine DNA

The level of 5′-methylcytosine in *O. viverrini* genomic DNA (gDNA) was determined by enzyme-linked immunosorbent assay using a 5-mC ELISA kit (Zymo Research, Irvine, CA, USA), as per the manufacturer’s protocol. The experiment was performed in 2 independent sets of *O. viverrini* gDNA and presented as the percentage of 5′-methylcytosine per total DNA.

### RNA extraction

Total RNA was extracted from worms using NucleoZOL, according to manufacturer’s protocol (Macherey-Nagel, Düren, Germany). Quantity, purity, integrity and potential contamination of the total RNA were determined by NanoDrop 2000 spectrophotometry (NanoDrop Technologies), agarose gel electrophoresis, Qubit 3.0 Fluorometer (Thermo Fisher Scientific), and Agilent 2100 Bioanalyzer (Agilent, Santa Clara, CA, USA). For eukaryotic organisms, mRNA was enriched from total RNA using oligo dT beads. rRNA from prokaryotic organisms was also removed using specialized kits. All retrieved mRNAs were randomly fragmented in fragmentation buffer, followed by cDNA synthesis.

### Library construction and sequencing

For each sample, sequencing libraries were generated, and RNA sequencing was performed according to the manufacturer’s protocol using a NEBNext^®^ Ultra^TM^ RNA Library Prep Kit for Illumina (Illumina^®^, NEB, Ipswich, MA, USA). The first-strand cDNA was generated using random hexamers and reverse transcriptase. After first-strand synthesis, the second strand was subsequently generated using RNase H and *Escherichia coli* polymerase I with dNTPs. The cDNA was then purified using AMPure XP beads (Beckman Coulter, Indianapolis, IN, USA), followed by a round of purification, terminal repair, A-tailing, ligation of sequencing adapters, size selection, and PCR enrichment. The final cDNA library concentration was quantified using a Qubit 2.0 fluorometer (Life Technologies, Carlsbad, CA, USA) and then diluted to 1 ng/μL before checking insert size on an Agilent Bioanalyzer 2100 system and quantifying to greater accuracy by quantitative PCR. Quality checked and equimolar pooled libraries were sequenced in an Illumina HiSeq system. Library construction and sequencing was performed by professional technicians of Novogene Co., Ltd. The raw RNA sequencing data have been deposited in the Sequencing Reads Archive (SRA) under accession number PRJNA1127773.

### Transcript assembly and gene functional annotation

Raw reads were quality checked and filtered to remove reads containing adapters or reads of low quality (poly-N-containing reads (*N* > 10%), and low-quality reads (quality score ≤ 20)) using the Illumina CASAVA package. The reads were mapped to the transcriptomes of *O. viverrini* (PRJNA222628) in *WormBase ParaSite* [16, 48]. Trinity [14] was utilized to complete the transcriptome reconstruction process with default parameters. Corset was applied to hierarchically cluster short transcripts into long genes for the downstream analysis [12]. For gene functional annotation, the assembled transcriptome was annotated using seven public databases as follows: NCBI non-redundant protein sequences, NCBI nucleotide sequences, InterPro (https://www.ebi.ac.uk/interpro/), COG (Cluster of Orthologous Groups of proteins) and KOG (euKaryotic Orthologous Groups) (KOG/COG), Swiss-Prot, Gene Ontology (GO), and KEGG (Kyoto Encyclopedia of Genes and Genome).

### Differential gene expression analysis (DEGs)

The *de novo* transcriptome, filtered by Corset, was used to quantify the expression levels using RSEM [23]. The count of clean reads for each transcript was determined and subsequently normalized to fragments per kilobase of exon model per million reads (FPKM), associating the read counts with the gene expression levels. The quantification of differentially expressed genes (DEGs) in each sample was calculated by DEseq2. Genes with adjusted *p*-value <0.01 [41] and log2 fold-difference >1 were regarded as significantly differently expressed.

### Gene annotation and gene ontology enrichment analysis

The sequences were annotated using Blast2GO [9] which produced combined graphical outputs for the three gene ontology (GO) categories: biological processes, molecular functions, and cellular components. Enrichment analysis of differentially expressed genes (DEGs) was performed using GOseq and topGO v2.38.1 [49]. The *p*-value was calculated in a hypergeometric test. GO vocabulary with corrected *p*-value <0.05 was significantly enriched in DEGs.

### Statistical analysis

To compare the two groups, nonparametric data were analyzed using the Kruskal–Wallis and Mann–Whitney U tests, while parametric data were analyzed using Student’s *t*-test or paired *t*-test. *p*-values of 0.05 or less were considered significant. All statistical analyses were performed using GraphPad Prism version 10 (GraphPad, La Jolla, CA, USA) or IBM SPSS Statistics version 22 for Mac (IBM Analytics, Armonk, NY, USA). All statistical tests for transcriptomics analysis were performed in R packages, as described above.

## Results

### Infection with *O. viverrini* lowering fasting blood glucose of infected diabetic hamsters

**Supplementary Figure S1:**
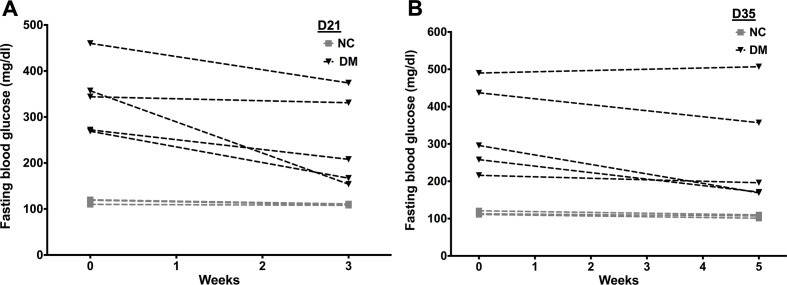
Comparison of fasting blood glucose levels in hamsters before *O. viverrini* infection and at necropsy. Individual data points represent each hamster’s glucose levels at the two time points.

To examine the effect of *O. viverrini* infection on blood glucose, fasting blood glucose (FBG) levels were measured before infection and during necropsy (Fig. 1B, Fig. S1A, and Fig. S1B). The results revealed that there were no changes in FBG in the NC group. Interestingly, these levels decreased moderately in DM hamsters after infection at both 3 (D21) and 5 (D35) weeks, based on a paired *t*-test comparing before and after infection for each hamster. However, the FBG levels in DM hamsters remained high, above the cut-off for diabetic diagnostic values (more than 126 mg/dL).

### Effects of diabetes on worm establishment, growth, and development

**Figure 2:**
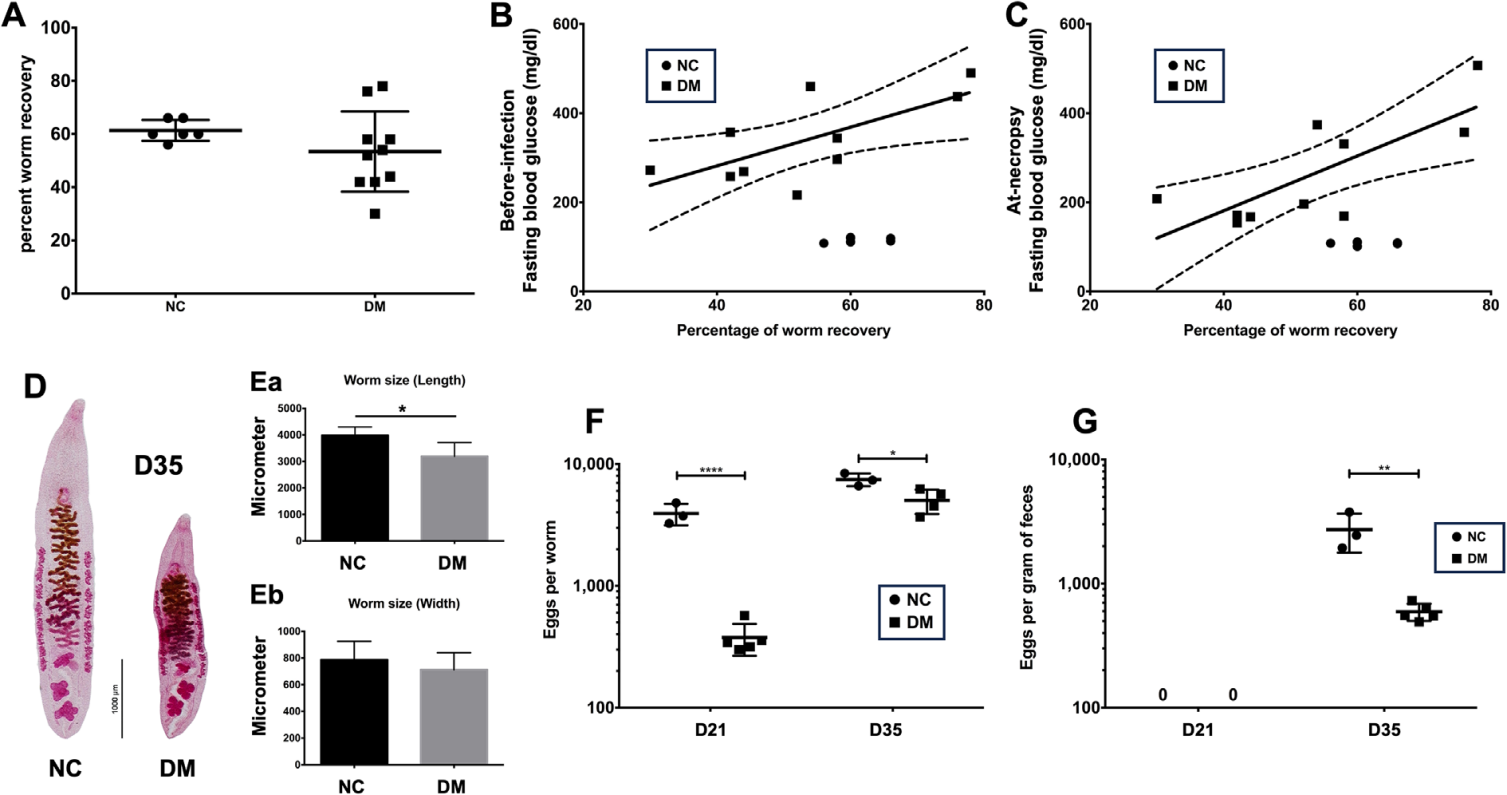
Parasitological studies of *O. viverrini* in diabetic hamsters. (A) Percentage of worm recovery between groups. (B and C) Correlation between fasting blood glucose levels and percent worm recovery. (D and E) Adult worm size comparison between groups. (F and G) Maturity of the reproductive system as shown in eggs per worm and eggs per gram of feces. ^*^p < 0.05, ^**^p < 0.01, ^***^p < 0.001, and ^****^p < 0.0001.

The worm establishment rate of *O. viverrini* was compared in diabetic hosts vs non-diabetic hosts. The infection rate of *O. viverrini* in diabetic hosts was not significantly different from that in normal hosts (Fig. 2A). Interestingly, the percentage of worm recovery in the DM group was positively correlated with both pre-infection and at-necropsy fasting blood glucose levels (*r* = 0.7348, *p* = 0.0379, and *r* = 0.8079, *p* = 0.0153, respectively) (Fig. 2B and Fig. 2C). These correlations were not observed in normal hosts (NC).

Due to the limited number of retrieved worms, worm staining and worm size measurements were conducted only for the D35 group. Compared to the worms from normal hosts, the adult worms from diabetic hosts were significantly shorter. However, in terms of width, the worms from each group were not different (Fig. 2D, Fig. 2Ea, and Fig. 2Eb).

The effect of the diabetic host on the maturity of the reproductive system, reflected by parasite egg production, was observed by using the number of eggs per worm and eggs per gram of feces. Eggs per worm in the DM group at both time points were lower than in the normal control group (Fig. 2F). The number of eggs from 3-week-old worms in the DM group was 10 times lower than in the normal group. For 5-week-old worms, a 50% reduction in egg number was observed in the DM group.

The presence of eggs in hamster feces was determined using a modified formalin-ethyl acetate concentration technique. At 3 weeks post-infection (D21), parasite eggs were not present in the feces of either group. At D35, the number of eggs in DM hamsters was 5 times lower than that in the NC group (Fig. 2G).

### Diabetic condition altering the level of 5′-methylcytosine of the worm

**Supplementary Figure S2:**
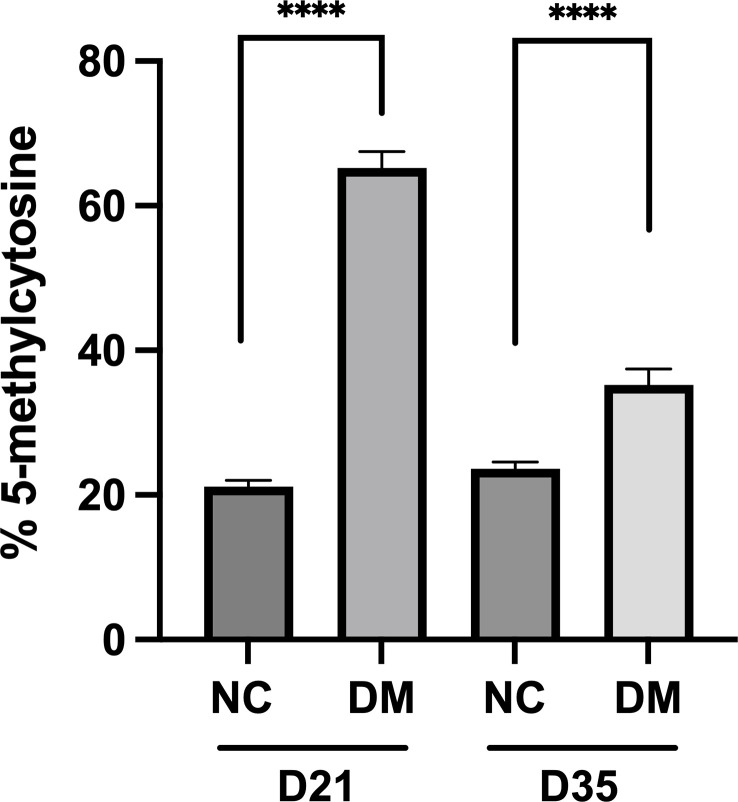
ELISA for 5′-methylcytosine for genomics DNA extracted from *O. viverrini* flukes from diabetic (DM) and non-diabetic (NC) hamsters at days 21 and 35 post-infection (D21 and D35, respectively). ^***^p < 0.001, and ^****^p < 0.0001.

To examine the effect of diabetes on the epigenetic regulation of the *O. viverrini* worm, we conducted an ELISA test to screen for levels of 5′-methylcytosine (5′mC), a classical epigenetic marker, on genomic DNA. The results showed increased genome-wide 5′mC levels in *O. viverrini* worms under diabetic conditions at both 3 and 5 weeks of age (Supplementary Fig. S2). This change in epigenetic regulation is likely related to transcriptome changes in the worm, as alterations in DNA methylation can influence gene expression patterns.

### Transcriptome profiles of *O. viverrini*

Four samples of fifty *O. viverrini*, recovered from normal control (NC) and diabetes mellitus (DM) hamsters at 21 and 35 days post-infection (D21 and D35), were sequenced individually using double-stranded cDNA (dscDNA) libraries generated from RNA transcripts. Each sample achieved Q20 and Q30 quality scores exceeding 90%. RNA-seq analysis of the transcriptomes yielded 43.91, 45.64, 41.59, and 44.14 million raw reads for the D21-NC, D21-DM, D35-NC, and D35-DM samples, respectively. After quality filtration, 6.4, 6.5, 6.0, and 6.4 gigabases of clean data were obtained for the respective samples. The clean reads were aligned to the *O. viverrini* reference genome (PRJNA222628) available in the WormBase ParaSite database. Approximately 44.44% of clean reads from all samples mapped uniquely to annotated protein-coding genes.

### Sample correlation analysis

**Figure 3:**
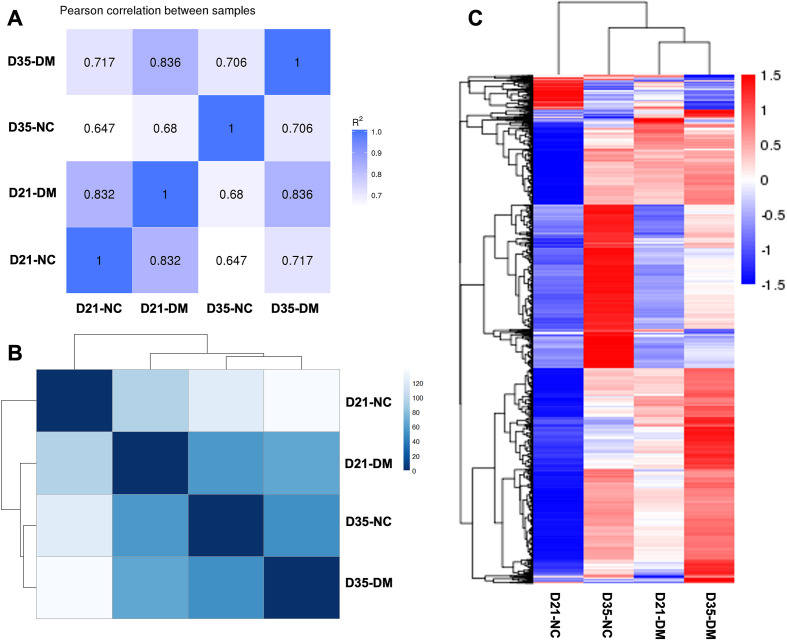
Sample relationships revealed by three different approaches. (A) Pearson’s correlation analysis between samples. The figures in the matrix *R*^2^ are the squares of the correlation coefficient (r) between two samples. (B) Clustering of samples using Sample Distance Matrix. (C) Hierarchical clustering showing divergent transcriptomic signatures among the four samples. The color scale indicates the Z-score values.

Pearson’s correlation analyses of the global expression profiles revealed substantial differences in transcript levels between worms of different ages and host conditions (hamsters with or without diabetes mellitus (DM vs NC)) (R² < 0.85). Interestingly, worms from diabetic hamsters exhibited the highest similarity in their expression profiles (*R*^2^ = 0.836) (Fig. 3A). This suggests that the diabetic condition of the host significantly influences the gene expression patterns of the fluke parasite. The hierarchical clustering analysis of the global expression profiles and the sample distance matrix further demonstrated distinct differences among the compared samples (Fig. 3B and Fig. 3C). Notably, the sample distance matrix indicated greater similarity in the magnitude of expression between different host conditions at 35 days compared to the D21 paired comparison.

### Differentially expressed genes

**Figure 4:**
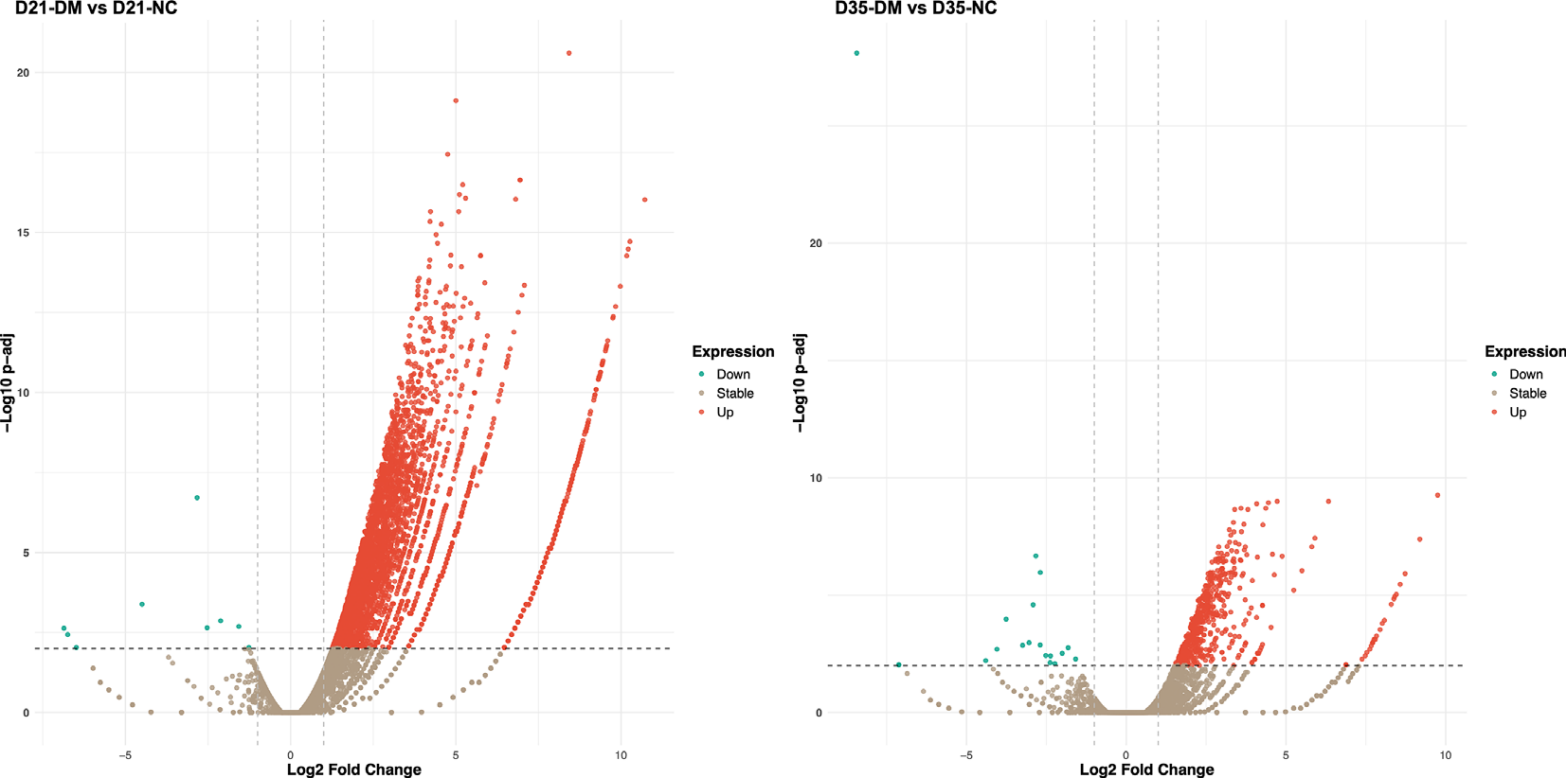
Volcano plots of differentially expressed genes (DEGs) in *O. viverrini* from diabetic (DM) and non-diabetic (NC) hamsters collected at days 21 and 35 post-infection (D21 and D35, respectively). The left panel shows the comparison between D21-DM and D21-NC, while the right panel shows the comparison between D35-DM and D35-NC. The red dots indicate upregulated genes, green dots indicate downregulated genes, and brown dots indicate genes with stable expression.

A total of 16,379 gene transcripts were detected using FPKM >0.01 as the threshold value, by cross-referencing the *O. viverrini* gene coordinates in the WormBase ParaSite database. Two independent pairwise comparisons between *O. viverrini* worms of the same age, recovered from non-diabetic control (NC) and diabetic mellitus (DM) hamsters, were performed to identify differentially expressed genes (DEGs). Significant differential expression was determined with a false discovery rate of 0.01 and a *q*-value (*p*-adjusted value) <0.01. Fig. 4A and Fig. 4B illustrate the distribution of up- and down-regulated genes in the paired comparisons. Comparing worms from NC and DM hamsters at 21 days post-infection, 4314 DEGs were identified, with 4305 up-regulated and 9 down-regulated mRNAs in D21-DM vs. D21-NC (Supplementary Table S1). In the comparison at 35 days post-infection, 567 DEGs were found, with 548 up-regulated and 19 down-regulated mRNAs in D35-DM vs. D35-NC (Supplementary Table S2). The vast difference in the number of DEGs between the D21 and D35 comparisons was evident. This finding correlates with the sample distance matrix and hierarchical clustering analysis of the global expression profiles, which indicated that the D35 pair is more similar than the D21 pair. The top ten up- and down-regulated genes (except for the nine down-regulated genes in the D21 comparison) are shown in Tables 1 and 2, respectively.

**Table 1:** List of the top 10 up-regulated and down-regulated DEGs from 21 days post-infected worms.

Gene ID	Log 2-fold changes	Protein (WormBase ParaSite)	Protein (UniProtKB)
Up-regulated genes			
T265_15648	10.71862214	C-Jun-amino-terminal kinase-interacting protein 4	Uncharacterized protein
T265_14061	10.26806659	WW domain-containing protein	Abnormal spindle-like microcephaly-associated protein ASH domain-containing protein
T265_04201	10.21595837	Huntingtin-interacting protein 1	I/LWEQ domain-containing protein
T265_03561	10.17104935	DUF4201 domain-containing protein	CCDC113/CCDC96 coiled-coil domain-containing protein
T265_14758	9.975974405	Clathrin-link domain-containing protein	Clathrin heavy chain linker core motif domain-containing protein
T265_10886	9.833383209	Cilia- and flagella-associated protein 57	Uncharacterized protein
T265_07118	9.762496418	Tektin	Tektin
T265_14131	9.750335781	CAP-Gly domain-containing linker protein 2	CAP-Gly domain-containing protein
T265_13290	9.595802803	Myoferlin	C2 domain-containing protein
T265_00579	9.568357921	LIM zinc-binding domain-containing protein	LIM zinc-binding domain-containing protein
Down-regulated genes			
T265_15558	−1.263433261	Transmembrane protein	Uncharacterized protein
T265_16061	−1.569051769	Aquaporin-3	Channel protein, MIP family
T265_15469	−2.119571346	CLU protein	Uncharacterized protein
T265_03259	−2.527706626	DUF3421 domain-containing protein	Uncharacterized protein
T265_09666	−2.830199546	ZZ-type domain-containing protein	Uncharacterized protein
T265_11697	−4.496302475	Peptidase C13 family protein	Peptidase C13 family protein
T265_12147	−6.485827838	N/A	Uncharacterized protein
T265_01861	−6.746176908	Fibronectin type-III domain-containing protein	Uncharacterized protein
T265_12806	−6.860619971	Secreted protein	Uncharacterized protein

**Table 2:** List of the top 10 up-regulated and down-regulated DEGs from 35 days post-infected worms.

Gene ID	Log 2-fold changes	Protein (WormBase ParaSite)	Protein (UniProtKB)
Up-regulated genes			
T265_02482	9.747656762	SCP domain-containing protein	SCP domain-containing protein
T265_08969	9.18902406	Transmembrane protein	Uncharacterized protein
T265_00952	8.728623988	Mitogen-activated protein kinase	Mitogen-activated protein kinase
T265_14885	8.574679766	SCP-like protein	SCP-like protein
T265_02307	8.453705352	N/A	Uncharacterized protein
T265_15112	8.402324675	C2H2-type domain-containing protein	C2H2-type domain-containing protein
T265_13831	8.375931425	Thyroid hormone receptor beta	Zinc finger, C4 type
T265_09416	8.2937247	Kinesin light chain	Kinesin light chain
T265_06842	8.0814622	N/A	Uncharacterized protein
T265_00244	8.014601607	DUF2326 domain-containing protein	Uncharacterized protein
Down-regulated genes			
T265_08985	−2.82986436	EF hand	EF-hand domain-containing protein
T265_15680	−2.915432056	SMB domain-containing protein	Uncharacterized protein
T265_02779	−3.042707987	Ig-like domain-containing protein	Uncharacterized protein
T265_03590	−3.24385967	N/A	Uncharacterized protein
T265_07366	−3.760252486	UPF0506 domain-containing protein	UPF0506 domain-containing protein
T265_01139	−4.048278263	Sorting nexin-14	Uncharacterized protein
T265_11858	−4.398980041	Pecanex-like protein	Uncharacterized protein
T265_02040	−7.118897827	N/A	Uncharacterized protein
T265_12390	−7.118897827	N/A	Uncharacterized protein
T265_14863	−8.435550979	Kinesin motor domain-containing protein	Uncharacterized protein

### Gene ontology enrichment

**Figure 5:**
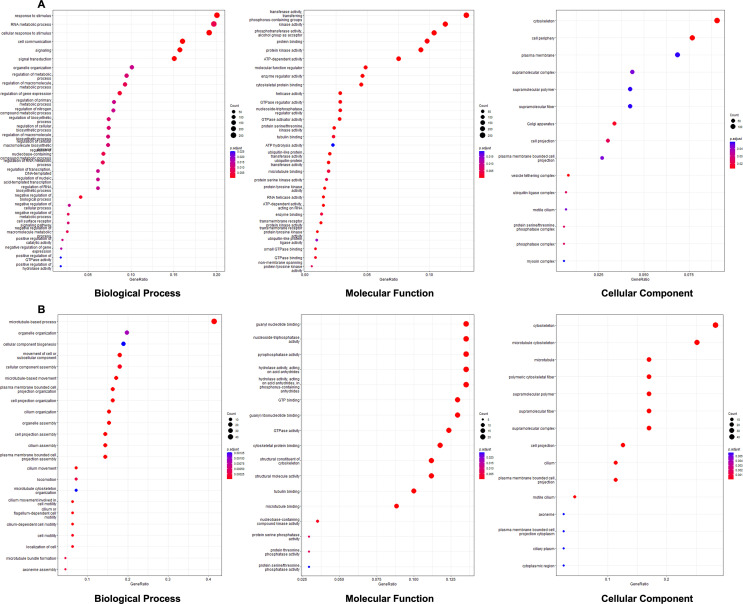
Gene Ontology enrichment analysis of *O. viverrini* collected at days 21 and 35 post-infection in diabetic (DM) and non-diabetic (NC) hamsters. (A) GO enrichment analysis at day 21 post-infection. (B) GO enrichment analysis at day 35 post-infection.

To elucidate the molecular adaptations of *O. viverrini* during infection in different host environments, gene ontology (GO) enrichment analysis was performed on transcriptomic data from worms collected at days 21 and 35 post-infection from both diabetic and non-diabetic hamsters. Fig. 5A and Supplementary Table S3 represent *O. viverrini* at 21 days post-infection. At this stage, the biological processes enriched in the parasite show a strong emphasis on “responses to stimuli”, “RNA metabolic processes”, and various regulatory mechanisms, including cell communication and signaling, reflecting the parasite’s active adaptation to its host environment through signaling and metabolic processes. The molecular functions are particularly enriched for transferase activity, various phosphorus-oxygen lyase activities, and enzyme regulator activities. The cellular components category at day 21 highlights terms like “cytoskeleton”, “plasma membrane”, and “supramolecular complex”, which are primarily membrane-associated, suggesting the parasite’s interface with the host environment and robust cellular machinery for parasite expansion.

**Supplementary Figure S3:**
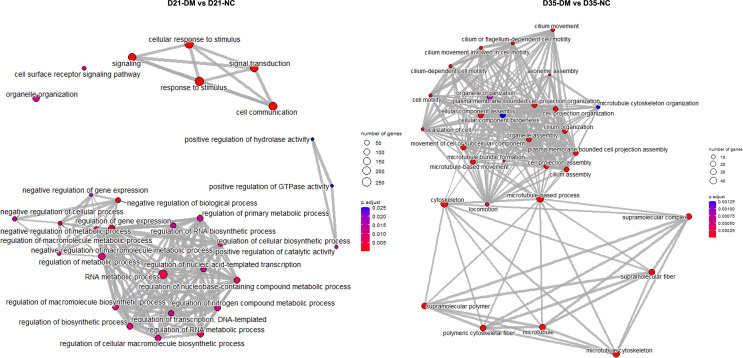
Gene Ontology enrichment mapping for DEGs in *O. viverrini* comparing between flukes from diabetic (DM) and non-diabetic (NC) hamsters at days 21 and 35 post-infection (D21 and D35, respectively). The left panel shows the comparison between D21-DM and D21-NC, while the right panel shows the comparison between D35-DM and D35-NC.

In contrast, at day 35 post-infection (Fig. 5B and Supplementary Table S4), the GO enrichment profile in the biological processes shifts towards cellular organization and assembly, including “microtubule-based process”, “organelle organization”, and “cellular component biogenesis”, implying a transition towards cellular maturation, structural remodeling, and differentiation in response to the host microenvironment. The molecular function category shows enrichment in terms like “guanyl nucleotide binding”, “nucleoside-triphosphatase activity”, “pyrophosphatase activity”, and “hydrolase activity”, indicating changes in energy metabolism and signal transduction. The cellular components enriched at this stage highlight “cytoskeleton”, “microtubule cytoskeleton”, “microtubule”, and “supramolecular polymer” as key terms, aligning with the observed shift in biological processes. The gene ontology enrichment mapping of both the D21 and D35 pair is shown in Supplementary Figure S3.

### Expression profile of hepatobiliary opisthorchiasis-related genes

Previous analyses in this study have predominantly highlighted genes related to growth, development, and response to stimuli in *O. viverrini*, reflecting the parasite’s adaptation to its host environment. However, genes directly associated with pathogenesis were not prominently featured in these initial results and have not yet been discussed. This is particularly intriguing given our prior findings that *O. viverrini* infection leads to more severe liver pathology in diabetic hamsters compared to non-diabetic controls (reference lacking here). To address this gap and further elucidate the molecular basis of enhanced pathogenicity in diabetic hosts, we examined the expression patterns of key genes known to be involved in *O. viverrini* virulence.

**Figure 6:**
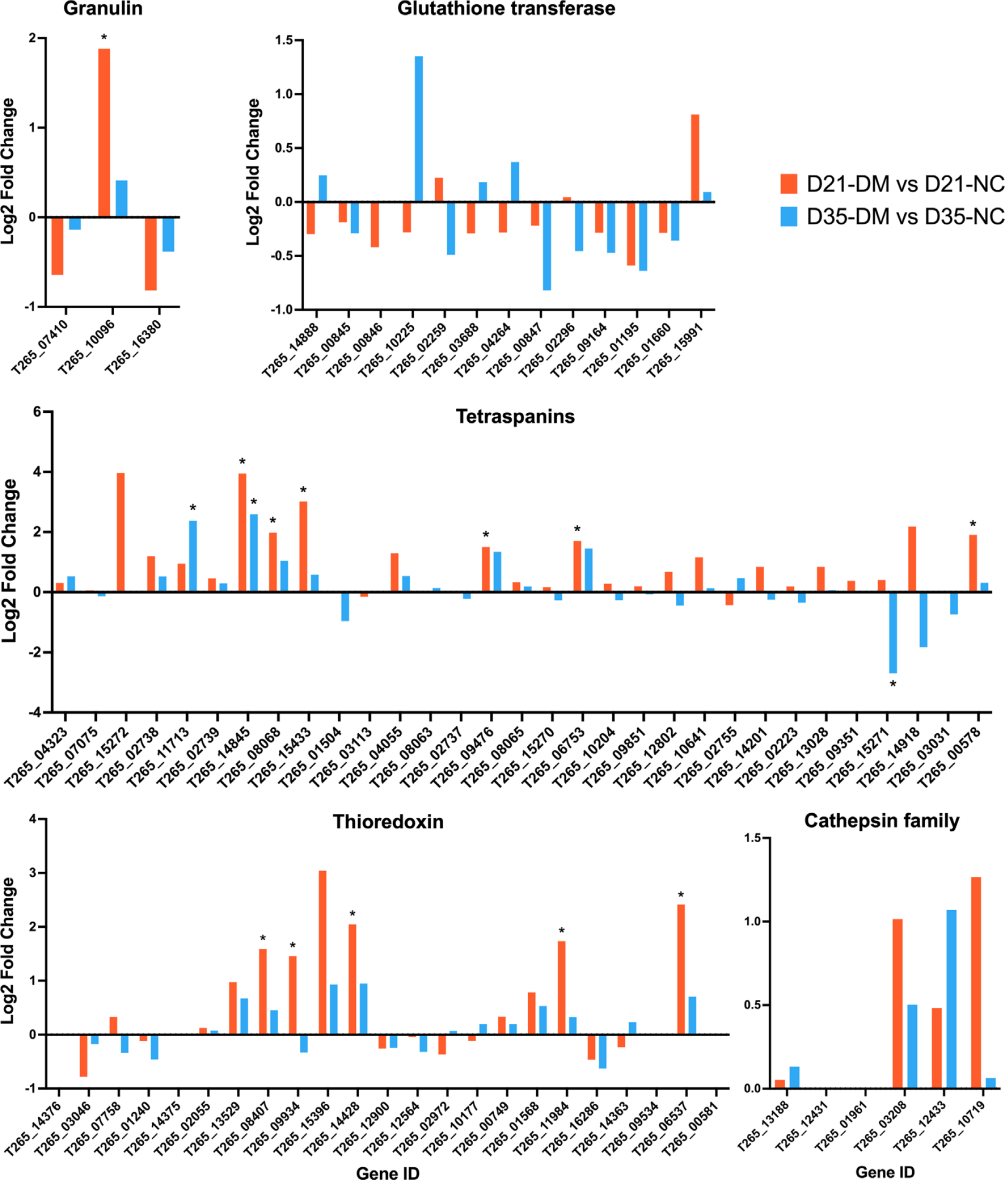
Differential expression of key gene families involved in the pathogenesis of *O. viverrini* infection. *Opisthorchis viverrini* flukes were collected from diabetic (DM) and non-diabetic (NC) hamsters at day 21 (D21) and day 35 (D35). The log2 fold change in expression levels is presented for granulin, glutathione transferase, tetraspanins, thioredoxin, and the cathepsin family genes. Orange bars represent the comparison between D21-DM and D21-NC, while blue bars represent the comparison between D35-DM and D35-NC. ^*^ indicates genes with significant differential expression (DEGs).

Figure 6 illustrates the differential expression of several gene families known to be involved in the pathogenesis of *O. viverrini* infection, comparing *O. viverrini* collected from diabetic (DM) and non-diabetic (NC) hamsters at days 21 and 35 post-infection (D21 and D35, respectively). These data are derived from the DEGs identified during transcriptome analysis. The genes depicted include granulin, glutathione transferase, tetraspanins, thioredoxin, and the cathepsin family.

The data, presented as log2 fold change, reveal distinct expression profiles across several important protein families. Granulin shows significant upregulation of one gene (T265_10096) in *O. viverrini* at D21, while glutathione transferase genes exhibit varied responses without any significant DEGs. Notably, tetraspanins demonstrate widespread upregulation, particularly at D21, with multiple genes showing statistically significant changes. This pattern persists at day 35, though the specific genes showing significant changes vary. Thioredoxin genes also display strong upregulation at D21, with several significant alterations. The cathepsin family genes trend towards upregulation, albeit with less pronounced changes, and no significant DEGs have been demonstrated.

## Discussion

Variations in host metabolism, genetic background, and immune responses significantly influence host-parasite interactions, affecting the parasite’s infection capacity, growth, development, reproductive maturity, and pathology within the host [1, 6, 18, 26]. This study examines the impact of diabetes, a chronic condition altering host metabolism and immune function, on the growth and development of the liver fluke *O. viverrini*. Additionally, we analyzed transcriptome changes between juvenile adult worms (21 days post-infection) and mature adult worms (35 days post-infection) in diabetic and non-diabetic hosts.

Our findings indicate that *O. viverrini* exhibits stunted development in diabetic hosts, evidenced by smaller size and reduced egg production. Interestingly, the infection rate of *O. viverrini* correlated positively with fasting blood glucose levels. This could be attributed to the impaired immune response in diabetic hosts, which may create a more conducive environment for the parasite [18]. Furthermore, increased infection rates lead to higher worm density in the liver, resulting in food competition and smaller worm sizes.

Parasitic infections are known to act as a protective factor against diabetes, potentially preventing its onset and improving blood glucose levels in affected individuals [17, 26, 42, 51]. In our study, we also observed that *O. viverrini* infection decreased fasting blood glucose levels in diabetic hamsters. However, while previous research indicates that fasting blood glucose remains elevated for an extended period following streptozotocin-induced diabetes in animal models [13], the absence of a control group of uninfected diabetic hamsters in our study limits our ability to conclusively attribute the observed glucose reduction to the parasitic infection.

Various studies have indicated that the growth of *O. viverrini* depends on multiple host factors, including steroid hormones, free fatty acids, and bile salts, which the worms cannot produce *de novo* [1]. Diabetes is known to alter fatty acid metabolism, bile acid composition, and hormone production [15, 39, 46]. These changes likely contribute to the observed variations in *O. viverrini* growth and development in our study. For example, a previous study demonstrated that oral prednisolone treatment in *O. viverrini*-infected hamsters resulted in larger adult worms, highlighting the impact of external hormones on parasite growth [21].

Interestingly, research on *Schistosoma mansoni* in diabetic hosts has shown increased egg production and worm deposition in the liver [1], contrasting with our findings for *O. viverrini*. Despite both being fluke species, these differences might stem from their distinct habitats and nutritional requirements. While *S. mansoni* benefits from the altered metabolic environment in diabetes, *O. viverrini* may face constraints due to its specific needs and the competitive environment created by increased infection rates.

Transcriptomic analysis is a powerful tool to uncover the landscape of expressed genes and can be used to compare the level of expression of each gene between groups [8, 19]. Several studies have been carried out on the transcriptome of *O. viverrini* by multiple methods and they have shown some essential genes of the parasites, but are still far from completion [19, 47]. This study provides novel insights into the epigenetic and transcriptomic changes of *O. viverrini* when subjected to a diabetic environment in its hamster host, revealing significant alterations in gene expression patterns that may contribute to the parasite’s adaptation and survival in a hyperglycemic environment. These changes likely have profound implications for the parasite’s biology and its interaction with the host.

Our ELISA screening of genome-wide 5′-methylcytosine (5′mC) levels in *O. viverrini* revealed increased DNA methylation in worms recovered from diabetic hosts. This epigenetic marker is known to play a crucial role in regulating gene expression [22] and likely plays a crucial role in driving changes in gene expression patterns observed in our transcriptomic analysis. Such epigenetic modifications may be a response to the altered metabolic environment in diabetic hosts [43], potentially providing the parasite with adaptive advantages or facilitating its survival and proliferation under these conditions. The relationship between host diabetic status and parasite epigenetic changes warrants further comprehensive investigation, as it may provide insights into the mechanisms of host-parasite interactions in metabolically altered environments.

The transcriptome profiles of *O. viverrini* demonstrated substantial differences between worms recovered from normal and diabetic hosts, as well as between different time points post-infection. Importantly, the highest similarity in expression profiles was observed between worms from diabetic hosts at different time points, suggesting that the diabetic condition exerts a strong and consistent influence on parasite gene expression in adaptation to the diabetic milieu [1, 27, 44]. This finding is consistent with the greater similarity observed in the sample distance matrix for the D35 pairs compared to the D21 pairs, indicating that the differences in the host’s metabolic state exert a more pronounced effect at earlier stages of infection.

The differential gene expression analysis revealed a striking contrast in the number of differentially expressed genes (DEGs) between the 21-day and 35-day post-infection comparisons. The diabetic condition led to considerable upregulation of genes in the parasite. At D21, a striking 4314 differentially expressed genes (DEGs) were identified. This number reduced markedly at D35, with 567 DEGs, suggesting that the early stages of infection in a diabetic host require more extensive transcriptional reprogramming. Moreover, the substantial reduction in DEGs at D35 suggests possible stabilization or an adaptation phase in the parasite’s gene expression profile as the infection progresses. This finding implies that the initial adaptation to the altered environment is a critical period for the parasite, potentially involving the activation of stress response pathways and metabolic adjustments [10, 28, 52].

The GO enrichment analysis provided insights into the biological processes and molecular functions affected by diabetes. At 21 days post-infection, the enriched GO terms in biological processes were predominantly associated with responses to stimuli, RNA metabolic processes, and regulatory mechanisms. The molecular functions enriched at this stage included activities related to transferases, phosphorus-oxygen lyases, and enzyme regulators. The emphasis on transferase activities and membrane-associated cellular components suggests that the parasite is actively modifying its interface with the host’s altered environment and adjusting its metabolic processes with robust metabolic and regulatory activity. By D35, the GO enrichment profile shifted towards cellular organization and assembly processes, such as microtubule-based processes and organelle organization, implying a transition towards maturation and structural remodeling [48]. This change in focus from immediate response to longer-term adaptation suggests that *O. viverrini* undergoes temporal progression in its response to the diabetic host environment. The enrichment of terms related to guanyl nucleotide binding and hydrolase activity further indicates ongoing adjustments in the parasite’s metabolic processes and signal transduction pathways, aligning with the observed shift towards cellular component biogenesis and structural integrity. These changes may reflect adaptations to altered nutrient availability in the diabetic host [10, 52, 53], potentially allowing the parasite to exploit the hyperglycemic environment for its benefit [1].

Our study extends previous findings by delving into the expression profiles of specific gene families implicated in the pathogenesis of *O. viverrini* infection. This examination provides a crucial link between our previous observations of more severe liver pathology in diabetic hamsters [5, 6] and the underlying molecular changes in the parasite. The transcriptomic data revealed significant changes in the expression of several key gene families associated with the pathogenicity of *O. viverrini*, including granulin, tetraspanins, glutathione transferase, cathepsin family, and thioredoxin genes [24]. Granulin, a known growth factor implicated in cell proliferation and inflammation, exhibited significant upregulation in one gene at day 21 post-infection (D21) in worms from diabetic hamsters. Granulin has been implicated in cell proliferation and wound healing, and its overexpression in *O. viverrini* has been associated with hepatobiliary cell proliferation and potential carcinogenesis [7]. The increased expression of granulin in the diabetic environment suggests that the parasite may be exploiting the altered host metabolism to enhance its proliferative and potentially carcinogenic effects. Moreover, it also suggests a potential role in exacerbating hepatic inflammation and tissue remodeling in diabetic hosts, contributing to the more severe liver pathology observed in previous study. The most striking observation is the widespread upregulation of tetraspanins, particularly at D21. The persistent upregulation at D35 highlights the importance of these proteins in the parasite’s ability to adapt and persist in the host environment. Tetraspanins are involved in various cellular processes, including cell adhesion, motility, and signal transduction [35]. Their significant upregulation in *O. viverrini* infecting diabetic hosts suggests enhanced host-parasite interactions, potentially facilitating more efficient nutrient acquisition or immune evasion. This could contribute to the observed increased pathogenicity in diabetic conditions. Thioredoxin genes displayed strong upregulation at D21, indicating their potential role in counteracting oxidative stress and maintaining redox homeostasis within the parasite [33]. This upregulation may be a response to the altered metabolic state in diabetic hosts, allowing *O. viverrini* to thrive despite the heightened oxidative stress damage associated with diabetes and sustain its survival and virulence. The expression levels of glutathione transferase genes, which are implicated in neutralizing reactive oxygen species and xenobiotics [11], and cathepsin family genes, proteases involved in diverse aspects of parasite biology including tissue invasion and immune evasion [31, 38], did not exhibit statistically significant changes. This suggests that while these genes are essential for parasite survival within the host, their expression may not be substantially influenced by the diabetic condition or could be regulated primarily through post-transcriptional mechanisms. These findings collectively paint a picture of *O. viverrini* adapting to the diabetic host environment through multiple molecular mechanisms. The upregulation of granulin, tetraspanins, and thioredoxin genes likely contributes to the observed increased pathogenicity and suggests that the parasite may be exploiting the altered metabolic and immune landscape in diabetic hosts to enhance its virulence and survival. These findings align with our previous observations of more severe liver pathology in diabetic hamsters, providing a molecular basis for the increased pathogenicity of *O. viverrini* in these hosts [5, 6].

The observed epigenetic and transcriptomic changes in *O. viverrini* under diabetic conditions highlight the intricate relationship between host metabolic status and parasite biology. The extensive transcriptional changes observed in *O. viverrini* within diabetic hosts suggest that the parasite possesses considerable plasticity in its gene expression programs [10, 25, 34], allowing it to thrive in metabolically altered environments. This adaptability may contribute to the persistence of *O. viverrini* infections and potentially exacerbate complications in diabetic patients, which could have implications for disease management and treatment strategies.

In conclusion, this study revealed the profound impact of host diabetic status on *O. viverrini*, demonstrating significant alterations in the parasite’s epigenetic and transcriptomic landscape. Our findings highlight the remarkable adaptability of *O. viverrini* to the altered metabolic and hormonal milieu of diabetic hosts, which can influence parasite development and pathogenicity. This transcriptomic analysis advances our understanding of host-parasite interactions in the context of metabolic disorders and provides compelling evidence for the complex interplay between diabetes and parasitic infections. Future research should focus on elucidating the specific mechanisms underlying these adaptations, particularly the roles of key differentially expressed genes identified in this study. Understanding these molecular interactions is crucial for developing targeted interventions, novel therapeutic approaches, and diagnostic tools for opisthorchiasis, especially in populations with a high prevalence of diabetes. These insights not only contribute to our fundamental knowledge of host-parasite dynamics, but also pave the way for improved management of parasitic infections in individuals with metabolic disorders like diabetes.

## Data Availability

The raw RNA sequencing data have been deposited on the Sequencing Reads Archive (SRA) under accession number PRJNA1127773.

## Supplementary material

The supplementary material for this article can be found at https://www.parasite-journal.org/10.1051/parasite/2024056/olm.

*Supplementary Table 1*: Comparison of *O. viverrini* from diabetic (DM) and non-diabetic (NC) hamsters at 21 days post-infection. *Supplementary Table 2*: Comparison of *O. viverrini* from diabetic (DM) and non-diabetic (NC) hamsters at 35 days post-infection. *Supplementary Table 3*: Gene Ontology enrichment profile in *O. viverrini* at 21 days post-infection. *Supplementary Table 4*: Gene Ontology enrichment profile in *O. viverrini* at 35 days post-infection.
